# 579 An Analysis of Burn ICU Days from the National Injury Resource Database

**DOI:** 10.1093/jbcr/irae036.213

**Published:** 2024-04-17

**Authors:** Anastasiya Ivanko, Jonathan E Schoen, William L Hickerson, Herb A Phelan, Bart D Phillips, Jeffrey E Carter, Denise Danos

**Affiliations:** University Medical Center (LSU Health), New Orleans, LA; University of Tennessee (Retired), Memphis, TN; Louisiana State University Health Sciences Center- New Orleans, New Orleans, LA; BData, Inc, Edina, MN; LSUHSC New Orleans, New Orleans, LA; University Medical Center (LSU Health), New Orleans, LA; University of Tennessee (Retired), Memphis, TN; Louisiana State University Health Sciences Center- New Orleans, New Orleans, LA; BData, Inc, Edina, MN; LSUHSC New Orleans, New Orleans, LA; University Medical Center (LSU Health), New Orleans, LA; University of Tennessee (Retired), Memphis, TN; Louisiana State University Health Sciences Center- New Orleans, New Orleans, LA; BData, Inc, Edina, MN; LSUHSC New Orleans, New Orleans, LA; University Medical Center (LSU Health), New Orleans, LA; University of Tennessee (Retired), Memphis, TN; Louisiana State University Health Sciences Center- New Orleans, New Orleans, LA; BData, Inc, Edina, MN; LSUHSC New Orleans, New Orleans, LA; University Medical Center (LSU Health), New Orleans, LA; University of Tennessee (Retired), Memphis, TN; Louisiana State University Health Sciences Center- New Orleans, New Orleans, LA; BData, Inc, Edina, MN; LSUHSC New Orleans, New Orleans, LA; University Medical Center (LSU Health), New Orleans, LA; University of Tennessee (Retired), Memphis, TN; Louisiana State University Health Sciences Center- New Orleans, New Orleans, LA; BData, Inc, Edina, MN; LSUHSC New Orleans, New Orleans, LA; University Medical Center (LSU Health), New Orleans, LA; University of Tennessee (Retired), Memphis, TN; Louisiana State University Health Sciences Center- New Orleans, New Orleans, LA; BData, Inc, Edina, MN; LSUHSC New Orleans, New Orleans, LA

## Abstract

**Introduction:**

Burns are a time-sensitive injury that require accurate assessment, triage, and treatment. Access to burn treatment has been a challenge since only 2% of hospitals in the United States have a burn center. To guide clinicians, the ABA created burn center referral guidelines. The objective of this study was to investigate the National Injury Resource Database (NIRD) and 2021 claims data to discern if patients with a primary diagnosis of burn injury and requiring ICU-level care were treated at burn centers.

**Methods:**

After obtaining IRB approval, NIRD, a validated composite of 53 databases representing Burn and Trauma centers in the United States, and de-identified 2021 claims data, which included hospitals, free-standing emergency rooms, Burn Centers (BC), and Trauma Centers (TC), were merged. Burn ICU days (BICUD) were used as opposed to admissions given the potential for interfacility transfers. Descriptive statistics were calculated using Excel, with logistic regression analysis of additional facility variables

**Results:**

Our study included 8,241 facilities with 615 TCs, and 134 BCs. Previous work has noted that 481 of the 615 TCs are NOT at facilities with a BC, and that 18 of the 134 BCs are NOT at facilities with a TC. Of the total 784,309 BICUD, 60.7% occurred at facilities without BCs or TCs and 39.3% occurred at facilities with either a TC or BC. 10.8% occurred at facilities with TC without a BC. 28.5% occurred at facilities with a BC, of which 87.2% occurred at facilities with TCs and 12.8% occurred at BCs without TCs.

**Conclusions:**

Our data shows that the majority of BICUD occur at facilities with neither BCs nor TCs. Patients requiring BICUD likely meet ABA referral criteria and if treated at non-BC, are not included in the Burn Care Quality Platform (BCQP). Additional studies are needed to discern the validity of the data, and the quantity/quality of the care occurring at all facilities, and if that care meets ABA referral criteria to a BC.

**Applicability of Research to Practice:**

This abstract sheds light on where the majority of burn patients are treated, and therefore will help pave the road to better understanding the quantity/quality burn care that occurs outside of burn and trauma centers.

